# A non-hydrostatic numerical model for simulating regular wave breaking and surf-swash zone motions

**DOI:** 10.1038/s41598-024-60470-3

**Published:** 2024-04-27

**Authors:** Ali Shirkavand, Kambiz Farrahi-Moghaddam

**Affiliations:** 1 Department of Civil Engineering, School of Basic Sciences, Varamin-Pishva Branch, Islamic Azad University, Varamin, Iran; 2 Department of Water Engineering, Faculty of Civil and Surveying Engineering, Graduate University of Advanced Technology, Kerman, Iran

**Keywords:** Ocean sciences, Civil engineering, Computational science

## Abstract

In the present study, a non-hydrostatic two-dimensional vertical model has been developed to simulate the breaking of regular waves and surf-swash zone motions on a sloping beach. The objective of the present study was to estimate parameters at depth. The governing equations, based on the pressure-correction projection method, were solved in two main phases. In the initial phase, intermediate velocities were acquired through the resolution of advection–diffusion and explicit dynamic pressure gradient terms within the momentum equations, employing a time-splitting technique. To ensure local momentum conservation and solution monotonicity, modifications were made to the governing equations and the solution approach. In the second phase, through the substitution of intermediate velocities and the corrected pressure gradient term from the momentum equations into the continuity equation, with the elimination of velocities, a Poisson pressure-correction equation was derived. In the discretization stage, an innovation was proposed to compute horizontal velocities at the locations where vertical velocities are present, significantly reducing computational costs. The equation was then converted into a system of linear equations, which was solved implicitly. Comparisons between numerical results and experiments concerning plunging and spilling breakers reveal that the developed model satisfactorily simulates the outcomes.

## Introduction

The phenomenon of wave breaking stands as one of the most intricate hydrodynamic processes, captivating the interest of numerous researchers over time. Despite the repeated experiments conducted over recent decades to understand various aspects of wave breaking, a comprehensive theory that can analyze the position of wave breaking and the post-breaking wave shape in all possible situations has not been presented so far.

The presented experimental and analytical methods primarily rely on phase-averaged parameters within the wave, including wave height, wavelength, and period. These methods are dedicated to characterizing these aspects in both regular and irregular waves at the breaking point. Phase-averaged methods are broadly categorized into two main groups. In the first category, the wave height at the breaking moment is obtained based on relationships, with their primary parameter being the ratio of breaking height to wave height in deep water. The development of this method was carried out by Munk^1^. In the second category of phase-integrated methods, when specific geometric proportions prevail on the wave profile, wave breaking occurs. The first description of this kind was provided by Stokes^2^. The mentioned methods are also classified into two categories based on the approach to extracting relationships. The first category is based on analytical relationships. The main assumption in these methods is that breaking occurs when the particle velocity, influenced by the wave field, exceeds the wave velocity. The most well-known equations in this category were presented by Michell^3^ and Miche^4^, utilizing linear wave theory. Another set of relationships is empirical and based on experimental results. The Kamphuis^5^ equation is an example of such relationships, used to determine the breaking point of waves and applicable to irregular waves as well.

As mentioned, the aforementioned methods only provide a general view of the point of wave breaking, offering no information about the characteristics of the wave during and after breaking. Another group of these methods also describes the characteristics of waves after breaking. Battjes and Jansen^6^ were able to develop relationships that calculate the wave height during and after breaking by combining the modified Miche^4^ criterion with the concept of energy dissipation due to hydraulic jump occurrence. Dally et al.^7^, by utilizing the concept of energy and the wave height analog at each depth, formulated a criterion to determine the wave breaking point and provided relationships that could predict the wave height after breaking. Although the phase-averaged methods can identify the general wave characteristics in the breaking zone, obtaining information about the temporal changes in bed shear stress, turbulence intensity, and flow velocity at depth is essential for more detailed studies on coastal currents and sediment transport. It is evident that since these methods rely on averaged wave parameters in the phase, their application in such cases is always associated with approximations and the acceptance of simplifying assumptions in the governing equations. Therefore, the development of numerical methods capable of expressing dynamic wave parameters at each moment becomes necessary.

In recent decades, with the rapid advancement of computer technology, models based on the numerical solution of equations governing wave propagation have rapidly developed. In this particular context, micro-scale models, originally designed for research applications, have experienced substantial growth. In these models, the tracking of the free surface involves methods like the Marker and Cell (MAC) method, the level set method, and the Volume of Fluid (VOF) method^8–12^. For example, in the study by Lin and Liu^13^, a model was introduced that is based on solving flow equations using the VOF method. These mentioned models are capable of simulating details of the wave profile before and during breaking, as well as flow details such as turbulence intensity and shear stresses. While these techniques can accurately describe wave overturning, they provide more details than coastal engineers practically need. Therefore, when these methods are used in coastal areas on a large scale, the computations significantly increase. Consequently, for large-scale and practical problems, faster and more efficient methods need to be sought.

In recent decades, certain researchers have successfully simulated wave breaking and run-up using non-hydrostatic models. The first attempt in this direction was made by Zijlema and Stelling^14^, who complemented the accurate and efficient model of Zijlema and Stelling^15^. They introduced a model that accurately simulates nearshore phenomena using only two layers. This model combines the momentum conservation method with the upwind depth estimation of water at locations corresponding to horizontal velocities in a staggered grid, following the approach proposed by Stelling and Duinmeijer^16^. Subsequently, Yamazaki et al.^17^ simulated wave breaking and run-up by incorporating the non-hydrostatic term into the equations of shallow water. They applied the upwind method to solve the continuity and momentum equations, employing a conservative approach. Ai and Jin^18^ discretized the advection terms of a non-hydrostatic, multi-layer model by employing a method based on momentum conservation. They introduced an innovative grid arrangement and an innovative wet-dry algorithm. This model can simulate the propagation, breaking, and run-up of waves. Generally, non-hydrostatic models with an appropriate number of vertical layers (10–20 layers) not only accurately determine wave breaking without any additional assumptions but can also be used to estimate parameters such as sediment concentration in depth in fluid-sediment simulations^19,20^. This is while, for simulating the propagation of wave breaking with strong nonlinear properties and high dispersion after breaking, a solution must be devised using a non-hydrostatic model employing a finite number of layers^21,22^. This is essential to identify the wave-breaking point and the accompanying dissipation of energy, integrating them into the main equations.

As the primary objective behind the development of non-hydrostatic models is to estimate parameters related to water surface elevation with minimal computational cost and a small number of layers^23^, researchers in this field have paid less attention to estimating the distribution of parameters in-depth^24,25^. However, it should be noted that this does not imply that non-hydrostatic models are incapable of estimating parameters such as velocity distribution in depth. To demonstrate this point, the present model has been developed to estimate the distribution of parameters in depth concerning wave breaking. Applying governing equations for simulating linear short wave propagation in deep and intermediate water or the propagation of long waves where nonlinear terms are less significant does not pose a problem. This is while the use of these equations in problems where nonlinear terms are more critical and the water surface in the boundary-fitted grid experiences more pronounced oscillations is associated with challenges such as discrepancies in phase and wave height. In some cases, it may not effectively simulate the propagation of such waves. The inefficiency of the equations in these simulations is attributed to the lack of solution monotonicity and the absence of local momentum conservation. In this study, to address this issue, a similar approach to that used in the scalar advection equations was adopted. Applying this method for the advection of the velocity vector quantity can alleviate the issues of non-monotonicity and lack of local momentum conservation in a non-hydrostatic model. Moreover, the developed model in this study has no restrictions on the number of layers, whether few or many. In recent decades, there has been a rising trend in employing the Navier–Stokes equations and their simplified forms, such as Bousinesque-type equations for simulating wave propagation^26–29^. Specifically, solving these equations through the finite volume method has gained prominence as an efficient approach^30–35^. This paper introduces a non-hydrostatic numerical model that solves the complete form of the two-dimensional Reynolds-averaged Navier–Stokes equations. The model utilizes the implicit finite volume method coupled with a Godunov-type shock-capturing scheme. In this study, the horizontal advective term is discretized and solved during a two-step explicit predictor–corrector algorithm. Furthermore, the exact Riemann solver is employed to calculate the relevant fluxes. The primary aim of the current research is to estimate parameters at depth using a non-hydrostatic model in scenarios of wave breaking where nonlinear terms are significant and the water surface experiences intense fluctuations. To achieve this objective, the present model was developed based on the Shirkavand and Badiei^22^ model. Increasing the number of layers is necessary for parameter estimation at depth, resulting in higher computational costs. Therefore, an innovation was proposed in the discretization stage of the current model to significantly reduce computational costs. Additionally, the MILUD preconditioner (Modified Incomplete LU factorization restricted to Diagonal) was utilized for computations, incurring lower computational costs compared to other preconditioners.

## Methods

### Governing equations

The governing equations for simulating free-surface flow consist of the two-dimensional vertical (2DV) Reynolds-averaged Navier–Stokes (RANS) equations in incompressible and non-steady form, accounting for (*P* = − *ρg*(*z* − *η*) + *ρq*)). These equations are expressed as:

1 $$\frac{\partial u}{{\partial x}} + \frac{\partial w}{{\partial z}} = 0$$

2 $$\frac{\partial u}{{\partial t}} + \frac{{\partial u^{2} }}{\partial x} + \frac{\partial uw}{{\partial z}} + g\frac{\partial \eta }{{\partial x}} + \frac{\partial q}{{\partial x}} = \,\frac{\partial }{\partial x}\left( {\nu_{h} \frac{\partial u}{{\partial x}}} \right) + \frac{\partial }{\partial z}\left( {\nu_{v} \frac{\partial u}{{\partial z}}} \right)$$

3 $$\frac{\partial w}{{\partial t}} + \frac{\partial wu}{{\partial x}} + \frac{{\partial w^{2} }}{\partial z} + \frac{\partial q}{{\partial z}} = \,\frac{\partial }{\partial x}\left( {\nu_{h} \frac{\partial w}{{\partial x}}} \right) + \frac{\partial }{\partial z}\left( {\nu_{v} \frac{\partial w}{{\partial z}}} \right)$$

Here, *P*, *ρ*, *g*, *η*, *t*, and *q* represent total pressure, water density, gravitational acceleration, water surface level, time, and dynamic pressure, respectively. The variables *w* and *u* denote velocity components in the *z* and *x* directions. The horizontal eddy viscosity (*ν*_*th*_) is obtained using the Smagorinsky^36^ method, while the vertical eddy viscosity (*ν*_*tv*_) is determined through the *k-ε* method. In the current model, the dimensionless Smagorinsky coefficient (*C*_*s*_) employed for computing *ν*_*th*_ has been set within the range of 0.1 to 0.2. Additionally, the adjustable constants *C*_*1ε*_, *C*_*2ε*_, *σ*_*k*_, *σ*_*ε*_, and *C*_*μ*_, which are associated with the production term, dissipation term, Prandtl number for turbulent kinetic energy, Prandtl number for turbulent dissipation rate, and eddy viscosity term in the *k-ε* turbulence model, have been set to 1.44, 1.92, 1, 1.3, and 0.09, respectively. It is important to highlight that the main techniques utilized in the current study are associated with the terms on the left-hand side of Eqs. (2) and (3). Henceforth, to streamline and simplify the equations and prevent unnecessary complexity, the presentation of terms on the right-hand side of these two equations will be omitted at different stages of the solution process. The vertical velocity at the water surface is determined by applying the kinematic boundary condition at the water surface:

4 $$\frac{\partial \eta }{{\partial t}} + u\frac{\partial \eta }{{\partial x}} = \left. w \right|_{z = \eta }$$

Considering the impermeability of the bed, the formulation of the kinematic boundary condition for calculating the normal velocity at the bed is as follows:

5 $$- u\frac{\partial h}{{\partial x}} = \left. w \right|_{z = - h}$$

Here, *h* represents the depth of still water. The dynamic boundary condition at the free surface is set with known air pressure, *P*_*a*_ = *0*. By equating the total pressure to the air pressure applied at the surface (*z* = *η*), the dynamic pressure at the surface is obtained:

6 $$P_{a} = - \rho_{0} g(z - \eta ) + \rho_{0} q \Rightarrow q = \frac{{P_{a} }}{{\rho_{0} }} \cong 0$$

where *ρ*_0_ is the density of water. In the 2DV model, the water surface elevation is one of the unknowns in the problem, calculated simultaneously with other unknown parameters. Through the integration of the continuity equation concerning the depth of the water and the application of the kinematic boundary conditions at the bed and free surface, the differential equation for the water surface elevation was derived:

7 $$\frac{\partial \eta }{{\partial t}} + \frac{\partial }{\partial x}\int_{ - h}^{\eta } {udz} = 0$$

### Grid system

**Figure 1:**
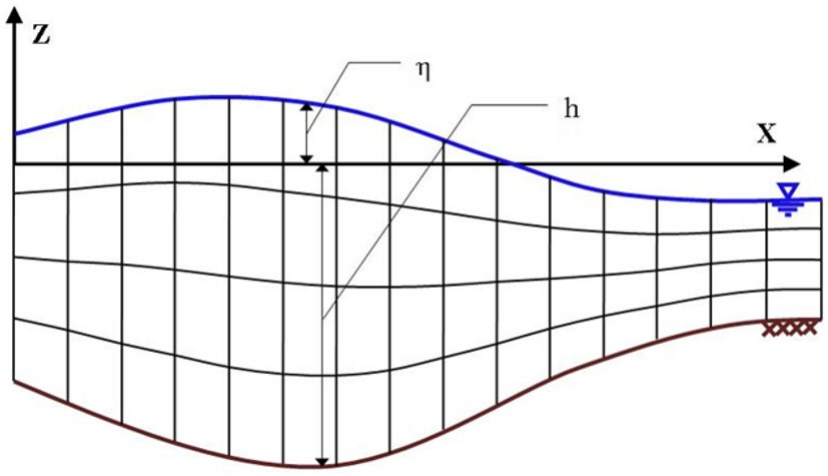
Computational grid layout with a curvilinear coordinate system aligned with the bed and water surface boundaries.

In this study, as depicted in Fig. 1, a boundary-fitted curvilinear grid was utilized, such that the surface and depth nodes were aligned with the water’s free surface and the bed, respectively. The vertical layer count remained consistent throughout the entire computational domain. The horizontal computational domain was partitioned into cells with varying dimensions (*∆x*). The total water depth (*D* = *h* + *η*) was segmented into several layers in this setup. The thickness of the layers (*d*_*k*_) was defined as a coefficient (*f*_*k*_) of the total water depth, i.e., *d*_*k*_ = *f*_*k*_·*D* = *f*_*k*_·*(η* + *h)*, where 0 < *f*_*k*_ < 1 and $\sum\limits_{k} {f_{k} = 1}$.

**Figure 2:**
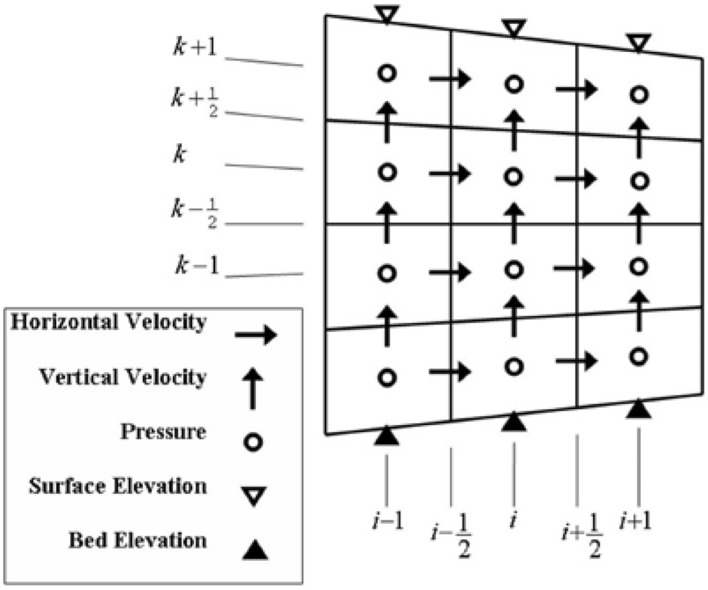
Schematic representation of variables in the computational grid.

The standard staggered grid system was employed to determine the locations of unknowns. Pressures were located at the cell centers (indices *i* and *k*), horizontal velocities at the right and left edges of cells (indices *i* + 1/2 and *k*), vertical velocities at the upper and lower edges of cells (indices *i* and *k* + 1/2), and the free surface elevation *η* and bed level -*h* at the upper and lower edges of each column and its center (index *i*) (Fig. 2).

### Discretization of governing equations and solution algorithm

Simulating phenomena with nonlinear behavior and significant dispersion necessitates employing a pressure correction projection approach, which has second-order temporal accuracy^14,15,18^. Consequently, the numerical technique adopted in this study involved the pressure correction projection method, which incorporates the elimination of velocities and a time-splitting technique^37^ for various solution stages. According to this approach, the equations were solved in two primary phases. In the first phase, the momentum equation, encompassing convection–diffusion terms, the water surface elevation gradient, and the explicit dynamic pressure gradient, was solved using a time-splitting algorithm and an appropriate solution approach for each part, yielding intermediate velocities. In the second phase, through the incorporation of intermediate velocities and the gradient of the corrected pressure term of the momentum equations into the continuity equation, thereby eliminating the velocities, the pressure correction equation—referred to as the Poisson equation—was obtained and solved. Finally, using the pressure correction values, the velocity values for the new time step were computed.

In recent years, the projection method coupled with the time-splitting technique in solving the Navier–Stokes equations has been widely used. Generally, this method significantly reduces computational complexities in multi-dimensional space and enhances the model's efficiency without compromising computational accuracy^38^. However, employing the time-splitting method in solving equations for the multidimensional advection of a scalar quantity in problems with free surface water fluctuations may encounter issues such as non-monotonicity^39^. To tackle this problem, solutions have been suggested in the literature, including the contributions of Easter^39^, Ruddick^40^, Wu and Falconer^41^, and others. It should be noted that in all the mentioned models, the basis of the applied modification is the same, such that the continuity equation and the advection equation are solved simultaneously in each direction. The differences lie only in the implementation and the method of solving the equations, which vary in terms of finite difference and finite volume. Additionally, all models proposed these modifications for the advection of scalar quantity. Regarding the propagation of long waves, whether assuming hydrostatic pressure or non-hydrostatic pressure, the advection equation does not require modification due to slight changes in the water surface^38,42–44^. Furthermore, since the modification performed relates to the advection term of the momentum equation and is insignificant in the linear shallow water wave propagation, the governing equations can simulate linear shallow water wave propagation in deep water without any alteration. However, in the problem under investigation in the present study, nonlinear terms are of great importance, and the computational grid, aligned with the water surface boundary, experiences more severe displacement. Consequently, the modeling conducted in this study encounters issues such as non-monotonicity in solutions and the lack of local momentum conservation, resulting in discrepancies in wave phase and height. Hence, in this study, to estimate parameters at depth and tackle the aforementioned issues using a non-hydrostatic model, a method akin to that employed in scalar advection equations was utilized.

The discretization of the momentum conservation equation involves integrating Eq. (1) vertically from *z*_*k-1/2*_ to *z*_*k*+*1/2*_, multiplying the result by *u*, and subtracting it from the integrated form of Eq. (2) within the specified layer:

8 $$\begin{aligned} & \frac{{u_{{i + \tfrac{1}{2},k}}^{n + 1} - u_{{i + \tfrac{1}{2},k}}^{n} }}{\Delta t} + \frac{1}{{d_{{i + \tfrac{1}{2},k}} }}\left( {\frac{{F_{i + 1,k}^{adv} - F_{i,k}^{adv} }}{{\Delta x_{{i + \tfrac{1}{2}}} }}} \right) - \,\,\frac{{u_{{i + \tfrac{1}{2},k}}^{n} }}{{d_{{i + \tfrac{1}{2},k}} }}\left( {\frac{{\phi_{i + 1,k} - \phi_{i,k} }}{{\Delta x_{{i + \tfrac{1}{2}}} }}} \right) + \,\,\left( {\frac{{\hat{u}_{{i + \tfrac{1}{2},k + \tfrac{1}{2}}} .\omega_{{i + \tfrac{1}{2},k + \tfrac{1}{2}}} - \hat{u}_{{i + \tfrac{1}{2},k - \tfrac{1}{2}}} .\omega_{{i + \tfrac{1}{2},k - \tfrac{1}{2}}} }}{{d_{{i + \tfrac{1}{2},k}} }}} \right) \\ & \quad - u_{{i + \tfrac{1}{2},k}}^{n} \left( {\frac{{\omega_{{i + \tfrac{1}{2},k + \tfrac{1}{2}}} - \omega_{{i + \tfrac{1}{2},k - \tfrac{1}{2}}} }}{{d_{{i + \tfrac{1}{2},k}} }}} \right) + g\frac{{\eta_{i + 1} - \eta_{i} }}{{\Delta x_{{i + \tfrac{1}{2}}} }} + \,\,\frac{1}{{d_{{i + \tfrac{1}{2},k}} }}\left( {\frac{{d_{i + 1,k} .q_{i + 1,k}^{n + \theta } - d_{i,k} .q_{i,k}^{n + \theta } }}{{\Delta x_{{i + \tfrac{1}{2}}} }}} \right) - \,\,\frac{{q_{{_{{i + \tfrac{1}{2},k + \tfrac{1}{2}}} }}^{n + \theta } }}{{d_{{i + \tfrac{1}{2},k}} }}.\frac{{Z_{{_{{i + 1,k + \tfrac{1}{2}}} }}^{{}} - Z_{{_{{i,k + \tfrac{1}{2}}} }}^{{}} }}{{\Delta x_{{i + \tfrac{1}{2}}} }} \\ & \quad + \frac{{q_{{_{{i + \tfrac{1}{2},k - \tfrac{1}{2}}} }}^{n + \theta } }}{{d_{{i + \tfrac{1}{2},k}} }}.\frac{{Z_{{_{{i + 1,k - \tfrac{1}{2}}} }}^{{}} - Z_{{_{{i,k - \tfrac{1}{2}}} }}^{{}} }}{{\Delta x_{{i + \tfrac{1}{2}}} }} = ... \\ \end{aligned}$$

Here, *n* is the time step index, and *F*^*adv*^ represents the flux arising from the advection of horizontal velocity in the *x*-direction, which will be discussed further. *φ*, *û* (advected horizontal velocity computed using the first order upwind method), *q*^*n*+*θ*^, and *ω* are also defined as follows:

9 $$\phi_{i,k} = \tfrac{1}{2}\left( {\phi_{{i + \tfrac{1}{2},k}} + \phi_{{i - \tfrac{1}{2},k}} } \right),\,\,\,\phi_{{i + \tfrac{1}{2},k}} = \hat{d}_{{i + \tfrac{1}{2},k}} .u_{{i + \tfrac{1}{2},k}}$$

10 $$\hat{u}_{{i + \tfrac{1}{2},k + \tfrac{1}{2}}} = \left\{ {\begin{array}{*{20}c} {u_{{i + \tfrac{1}{2},k}}^{{}} \,\,\,\,\,\,\,\,\,\,\,\,\,\,{\text{if}}\,\,\,\,\,\,\,\,\omega_{{i + \tfrac{1}{2},k + \tfrac{1}{2}}} > 0} \\ {u_{{i + \tfrac{1}{2},k + 1}}^{{}} \,\,\,\,\,\,\,\,\,\,{\text{if}}\,\,\,\,\,\,\,\,\omega_{{i + \tfrac{1}{2},k + \tfrac{1}{2}}} < 0} \\ \end{array} } \right.$$

11 $$q^{n + \theta } = \theta .q^{n + 1} + (1 - \theta ).q^{n} = \,\,q^{n} + \theta .(q^{n + 1} - q^{n} ) = q^{n} + \theta .\Delta q$$

12 $$\omega_{{i + \tfrac{1}{2},k + \tfrac{1}{2}}} = \tfrac{1}{2}(\omega_{{i + 1,k + \tfrac{1}{2}}} + \omega_{{i,k + \tfrac{1}{2}}} )\,\,,\,\omega_{{i,k + \tfrac{1}{2}}} = w_{{i,k + \tfrac{1}{2}}} - \frac{{\partial z_{{i,k + \tfrac{1}{2}}} }}{\partial t} - u_{{i,k + \tfrac{1}{2}}} \frac{{\partial z_{{i,k + \tfrac{1}{2}}} }}{\partial x}$$

$\hat{d}_{{i + \tfrac{1}{2},k}}$ represents the thickness of the k-layer at the cell edge, *q*^*n*^ is the value of the dynamic pressure from the preceding time step, *∆q* represents the pressure correction term, and *θ* represents the parameter associated with temporal discretization. In this study, the implicit Euler method was employed (*θ* = 1).

In simulating the propagation of waves with high nonlinearity, especially during wave breaking, the momentum equation’s advection terms play a crucial role, and the solution strategy significantly affects the accuracy and precision of the results. Among the four advection terms in the momentum equations, the horizontal velocity advection in the *x*-direction is more critical than the others. Therefore, the solution approach for this term differs from other advection terms. In the current study, ensuring temporal second-order accuracy involved employing a predictor–corrector method. In the prediction stage, the fluxes of the advection term were calculated using the original Godunov algorithm with an exact solution for the Riemann initial values presented by Toro^45^. In Toro^45^'s study, the Godunov-type scheme was devised initially for the one-dimensional scenario, yet its extension to two-dimensional and three-dimensional cases is straightforward. Additionally, given that in the current model, a time-splitting algorithm is utilized to address various equations, it necessitates the independent treatment of the horizontal velocity advection term along the horizontal direction. This approach facilitates the utilization of the analytical solution of the Riemann problem for flux calculations. Consequently, the implementation of the Godunov-type shock capturing scheme employing the Riemann problem solution for this term poses no significant challenges.

In the correction stage, a backward method was used. For the other advection terms, a simple upwind method was applied. In the prediction stage, the advection term of the horizontal momentum equation was discretized as follows:

13 $$\frac{{u_{{i + \tfrac{1}{2},k}}^{p} - u_{{i + \tfrac{1}{2},k}}^{n} }}{\Delta t} + \frac{1}{{d_{{i + \tfrac{1}{2},k}} }}\frac{{F_{i + 1,k}^{p - adv} - F_{i,k}^{p - adv} }}{{\Delta x_{{i + \tfrac{1}{2}}} }} = 0$$

where $u_{i + 1/2,k}^{p}$ is the calculated velocity in the prediction stage and $F_{i,k}^{p - adv}$ is the calculated value of the flux passing through the cell face in the prediction stage, which is computed using the Godunov method with the solution of the Riemann problem:

14 $$F_{i,k}^{p - adv} = \left\{ {\begin{array}{*{20}l} {\hat{d}_{{i - \tfrac{1}{2},k}} u_{L}^{2} \,\,\,\,\,\,\,\,\,\,\,\,\,\,\,\,\,\,\,\,\,\,\,\,\,\,\,\,\,\,{\text{if}}\,\,u_{L} > u_{R} \,,\,u_{L} + u_{R} > 0} \hfill \\ {\hat{d}_{{i - \tfrac{1}{2},k}} u_{L}^{2} = \,\,\hat{d}_{{i + \tfrac{1}{2},k}} u_{R}^{2} \,\,\,\,\,\,\,\,\,{\text{if}}\,\,u_{L} > u_{R} \,,\,u_{L} + u_{R} = 0} \hfill \\ {\hat{d}_{{i + \tfrac{1}{2},k}} u_{R}^{2} \,\,\,\,\,\,\,\,\,\,\,\,\,\,\,\,\,\,\,\,\,\,\,\,\,\,\,\,\,\,{\text{if}}\,\,u_{L} > u_{R} \,,\,u_{L} + u_{R} < 0} \hfill \\ {\hat{d}_{{i - \tfrac{1}{2},k}} u_{L}^{2} = \,\,\hat{d}_{{i + \tfrac{1}{2},k}} u_{R}^{2} \,\,\,\,\,\,\,\,{\text{if}}\,\,u_{L} = u_{R} } \hfill \\ {\hat{d}_{{i - \tfrac{1}{2},k}} u_{L}^{2} \,\,\,\,\,\,\,\,\,\,\,\,\,\,\,\,\,\,\,\,\,\,\,\,\,\,\,\,\,\,{\text{if}}\,\,u_{L} < u_{R} \,,\,u_{L} > 0} \hfill \\ {0\,\,\,\,\,\,\,\,\,\,\,\,\,\,\,\,\,\,\,\,\,\,\,\,\,\,\,\,\,\,\,\,\,\,\,\,\,\,\,\,\,\,{\text{if}}\,\,u_{L} < 0\,,u_{R} > 0} \hfill \\ {\hat{d}_{{i + \tfrac{1}{2},k}} u_{R}^{2} \,\,\,\,\,\,\,\,\,\,\,\,\,\,\,\,\,\,\,\,\,\,\,\,\,\,\,\,\,{\text{if}}\,\,u_{L} < u_{R} \,,\,u_{R} < 0} \hfill \\ \end{array} } \right.$$

Here, $u_{R}$ and $u_{L}$ are the initial values for the conserved variables at the right and left sides of the computational cell wall, respectively. After calculating $F_{i,k}^{p - adv}$ and substituting it into Eq. (13) to obtain $u_{i + 1/2,k}^{p}$ ‘ the final correction stage yields the flux due to the advection of horizontal velocity in the *x*-direction:

15 $$F_{i,k}^{adv} = \left\{ {\begin{array}{*{20}l} {\hat{d}_{{i + \tfrac{1}{2},k}} .(u_{{i + \tfrac{1}{2},k}}^{p} )^{2} \,\,\,\,\,\,\,\,\,\,{\text{if}}\,u_{{i + \tfrac{1}{2},k}} + u_{{i - \tfrac{1}{2},k}} \ge 0} \hfill \\ {\hat{d}_{{i - \tfrac{1}{2},k}} .(u_{{i - \tfrac{1}{2},k}}^{p} )^{2} \,\,\,\,\,\,\,\,\,\,{\text{if}}\,u_{{i + \tfrac{1}{2},k}} + u_{{i - \tfrac{1}{2},k}} < 0} \hfill \\ \end{array} } \right.$$

Similarly, the discretization of the conservative momentum equation in the vertical direction is as follows:

16 $$\begin{aligned} & \frac{{w_{{i,k + \tfrac{1}{2}}}^{n + 1} - w_{{i,k + \tfrac{1}{2}}}^{n} }}{\Delta t} + \,\,\frac{1}{{d_{{i,k + \tfrac{1}{2}}} }}\left( {\frac{{\hat{w}_{{i + \tfrac{1}{2},k + \tfrac{1}{2}}} \phi_{{i + \tfrac{1}{2},k + \tfrac{1}{2}}} - \hat{w}_{{i - \tfrac{1}{2},k + \tfrac{1}{2}}} \phi_{{i - \tfrac{1}{2},k + \tfrac{1}{2}}} }}{{\Delta x_{i} }}} \right) - \,\,\frac{{w_{{i,k + \tfrac{1}{2}}}^{n} }}{{d_{{i,k + \tfrac{1}{2}}} }}\left( {\frac{{\phi_{{i + \tfrac{1}{2},k + \tfrac{1}{2}}} - \phi_{{i - \tfrac{1}{2},k + \tfrac{1}{2}}} }}{{\Delta x_{i} }}} \right) \\ & \quad + \left( {\frac{{\hat{w}_{i,k + 1} \omega_{i,k + 1} - \hat{w}_{i,k} \omega_{i,k} }}{{d_{{i,k + \tfrac{1}{2}}} }}} \right) - \,\,w_{{i,k + \tfrac{1}{2}}}^{n} \left( {\frac{{\omega_{i,k + 1} - \omega_{i,k} }}{{d_{{i,k + \tfrac{1}{2}}} }}} \right) + \frac{{q_{i,k + 1}^{n + \theta } - q_{i,k}^{n + \theta } }}{{d_{{i,k + \tfrac{1}{2}}} }} = ... \\ \end{aligned}$$

Here, *ŵ* is the advected vertical velocity employing a first-order accuracy upwind method:

17 $$\hat{w}_{{i + \tfrac{1}{2},k + \tfrac{1}{2}}} = \left\{ {\begin{array}{*{20}c} {w_{{i,k + \tfrac{1}{2}}}^{{}} \,\,\,\,\,\,\,\,\,\,\,\,\,\,\,{\text{if}}\,\,\,\,\,\,\,\,\phi_{{i + \tfrac{1}{2},k + \tfrac{1}{2}}} > 0} \\ {w_{{i + 1,k + \tfrac{1}{2}}}^{{}} \,\,\,\,\,\,\,\,\,\,{\text{if}}\,\,\,\,\,\,\,\,\phi_{{i + \tfrac{1}{2},k + \tfrac{1}{2}}} < 0} \\ \end{array} } \right.$$

18 $$\hat{w}_{i,k + 1} = \left\{ {\begin{array}{*{20}c} {w_{{i,k - \tfrac{1}{2}}}^{{}} \,\,\,\,\,\,\,\,\,\,\,{\text{if}}\,\,\,\,\,\,\,\,\omega_{i,k + 1} > 0} \\ {w_{{i,k + \tfrac{1}{2}}}^{{}} \,\,\,\,\,\,\,\,\,\,{\text{if}}\,\,\,\,\,\,\,\,\omega_{i,k + 1} < 0} \\ \end{array} } \right.$$

The discretization of the equations as mentioned above ensures the mass and momentum conservation both globally and locally and guarantees monotonicity in solution of the nonlinear wave propagation in shallow water, along with phenomena such as wave breaking.

Furthermore, the discretization of the mass equation for the water column (Eq. 7) was as follows:

19 $$\frac{{\eta_{i}^{n + 1} - \eta_{i}^{n} }}{\Delta t} + \frac{{\hat{D}_{{i + \tfrac{1}{2}}}^{n} .U_{{i + \tfrac{1}{2}}}^{n + \theta } - \hat{D}_{{i - \tfrac{1}{2}}}^{n} .U_{{i - \tfrac{1}{2}}}^{n + \theta } }}{{\Delta x_{i} }} = 0\,$$

Here, $\hat{D}$ is the depth of the water at the cell edge, and $U_{{i + \tfrac{1}{2}}} = \sum\nolimits_{k} {f_{k} .u_{{i + \tfrac{1}{2},k}} }$ is the depth-averaged velocity. Calculating $\hat{D}$ using the upwind method and the equation proposed by Zijlema et al.^46^ enables the incorporation of wet/dry capability into the model.

As previously noted, the solution algorithm employed in the present study is rooted in a projection method with a pressure-correction technique. As an initial stage of this algorithm, the intermediate vertical and horizontal velocities (w* and u*) are calculated using the vertical and horizontal momentum equations, respectively. It is worth noting that this stage involves two components. At the first component of this stage, the terms related to dynamic pressure gradient were explicitly considered in the computations, using the values calculated at the previous time:

20 $$\frac{{u_{{i + \tfrac{1}{2},k}}^{*} - u_{{i + \tfrac{1}{2},k}}^{n} }}{\Delta t} + ... + \frac{{\partial q^{n} }}{\partial x} = ...$$

21 $$\frac{{w_{{i,k + \tfrac{1}{2}}}^{*} - w_{{i,k + \tfrac{1}{2}}}^{n} }}{\Delta t} + ... + \frac{{\partial q^{n} }}{\partial x} = ...$$

within the second component of this stage, the remaining pressure correction gradient term is employed, leading to the following equations:

22 $$\frac{{u_{{i + \tfrac{1}{2},k}}^{n + 1} - u_{{i + \tfrac{1}{2},k}}^{*} }}{\Delta t} + \theta \frac{{\partial \left( {\Delta q} \right)}}{\partial x} = 0$$

23 $$\frac{{w_{{i,k + \tfrac{1}{2}}}^{n + 1} - w_{{i,k + \tfrac{1}{2}}}^{*} }}{\Delta t} + \theta \frac{{\partial \left( {\Delta q} \right)}}{\partial z} = 0$$

In the second stage, through the substitution of the aforementioned equations into the mass equation, velocities are eliminated, and the Poisson equation for pressure correction, to calculate *∆q*, is obtained:

24 $$\begin{aligned} & \left[ { - \frac{{A_{{i - \tfrac{1}{2},k}}^{1} }}{{\Delta x_{i} }} + \frac{{S_{{i,k - \tfrac{1}{2}}}^{x} }}{{d_{{i,k - \tfrac{1}{2}}} }}B_{{i,k - \tfrac{1}{2}}}^{1} } \right](\Delta q)_{i - 1,k - 1} + \left[ { - \frac{{A_{{i - \tfrac{1}{2},k}}^{2} }}{{\Delta x_{i} }} - \frac{{S_{{i,k + \tfrac{1}{2}}}^{x} }}{{d_{{i,k + \tfrac{1}{2}}} }}B_{{i,k + \tfrac{1}{2}}}^{1} + \frac{{S_{{i,k - \tfrac{1}{2}}}^{x} }}{{d_{{i,k - \tfrac{1}{2}}} }}B_{{i,k - \tfrac{1}{2}}}^{2} } \right](\Delta q)_{i - 1,k} \\ & \quad + \left[ { - \frac{{A_{{i - \tfrac{1}{2},k}}^{3} }}{{\Delta x_{i} }} - \frac{{S_{{i,k + \tfrac{1}{2}}}^{x} }}{{d_{{i,k + \tfrac{1}{2}}} }}B_{{i,k + \tfrac{1}{2}}}^{2} } \right](\Delta q)_{i - 1,k + 1} + \left[ {\frac{{A_{{i + \tfrac{1}{2},k}}^{1} - A_{{i - \tfrac{1}{2},k}}^{4} }}{{\Delta x_{i} }} + \frac{{S_{{i,k - \tfrac{1}{2}}}^{x} }}{{d_{{i,k - \tfrac{1}{2}}} }}B_{{i,k - \tfrac{1}{2}}}^{3} + \frac{1}{{d_{{i,k - \tfrac{1}{2}}} }}} \right](\Delta q)_{i,k - 1} \\ & \quad + \left[ {\frac{{A_{{i + \tfrac{1}{2},k}}^{2} - A_{{i - \tfrac{1}{2},k}}^{5} }}{{\Delta x_{i} }} - \frac{{S_{{i,k + \tfrac{1}{2}}}^{x} }}{{d_{{i,k + \tfrac{1}{2}}} }}B_{{i,k + \tfrac{1}{2}}}^{3} + \frac{{S_{{i,k - \tfrac{1}{2}}}^{x} }}{{d_{{i,k - \tfrac{1}{2}}} }}B_{{i,k - \tfrac{1}{2}}}^{4} - \frac{1}{{d_{{i,k + \tfrac{1}{2}}} }} - \frac{1}{{d_{{i,k - \tfrac{1}{2}}} }}} \right](\Delta q)_{i,k} \\ & \quad + \left[ {\frac{{A_{{i + \tfrac{1}{2},k}}^{3} - A_{{i - \tfrac{1}{2},k}}^{6} }}{{\Delta x_{i} }} - \frac{{S_{{i,k + \tfrac{1}{2}}}^{x} }}{{d_{{i,k + \tfrac{1}{2}}} }}B_{{i,k + \tfrac{1}{2}}}^{4} + \frac{1}{{d_{{i,k + \tfrac{1}{2}}} }}} \right](\Delta q)_{i,k + 1} + \left[ {\frac{{A_{{i + \tfrac{1}{2},k}}^{4} }}{{\Delta x_{i} }} + \frac{{S_{{i,k - \tfrac{1}{2}}}^{x} }}{{d_{{i,k - \tfrac{1}{2}}} }}B_{{i,k - \tfrac{1}{2}}}^{5} } \right](\Delta q)_{i + 1,k - 1} \\ & \quad + \left[ {\frac{{A_{{i + \tfrac{1}{2},k}}^{5} }}{{\Delta x_{i} }} - \frac{{S_{{i,k + \tfrac{1}{2}}}^{x} }}{{d_{{i,k + \tfrac{1}{2}}} }}B_{{i,k + \tfrac{1}{2}}}^{5} + \frac{{S_{{i,k - \tfrac{1}{2}}}^{x} }}{{d_{{i,k - \tfrac{1}{2}}} }}B_{{i,k - \tfrac{1}{2}}}^{6} } \right](\Delta q)_{i + 1,k} + \left[ {\frac{{A_{{i + \tfrac{1}{2},k}}^{6} }}{{\Delta x_{i} }} - \frac{{S_{{i,k + \tfrac{1}{2}}}^{x} }}{{d_{{i,k + \tfrac{1}{2}}} }}B_{{i,k + \tfrac{1}{2}}}^{6} } \right](\Delta q)_{i + 1,k + 1} \\ & = \frac{1}{\theta \cdot \Delta t}\left[ {\frac{{d_{{i + \tfrac{1}{2},k}} }}{{\Delta x_{i} }}u_{i + 1/2,k}^{*} - \frac{{d_{{i - \tfrac{1}{2},k}} }}{{\Delta x_{i} }}u_{i - 1/2,k}^{*} - S_{{i,k + \tfrac{1}{2}}}^{x} u_{i,k + 1/2}^{*} + S_{{i,k - \tfrac{1}{2}}}^{x} u_{i,k - 1/2}^{*} + w_{i,k + 1/2}^{*} - w_{i,k - 1/2}^{*} } \right] \\ \end{aligned}$$

In which the coefficients *A*, *B*, and *S*^*x*^ are defined as:

25 $$\begin{aligned} A_{{i + \tfrac{1}{2},k}}^{1} & = \frac{{z_{{i + 1,k - \tfrac{1}{2}}} - z_{{i,k - \tfrac{1}{2}}} }}{{\Delta x_{{i + \tfrac{1}{2}}} }}g_{{i + \tfrac{1}{2},k - \tfrac{1}{2}}}^{1} \,,\,\,\,\,\,\,\,\,\,\,\,\,\,\,\,\,\,\,A_{{i + \tfrac{1}{2},k}}^{3} = - \frac{{z_{{i + 1,k + \tfrac{1}{2}}} - z_{{i,k + \tfrac{1}{2}}} }}{{\Delta x_{{i + \tfrac{1}{2}}} }}g_{{i + \tfrac{1}{2},k + \tfrac{1}{2}}}^{2} \\ A_{{i + \tfrac{1}{2},k}}^{2} & = - \frac{{d_{i,k} }}{{\Delta x_{{i + \tfrac{1}{2}}} }} + \frac{{z_{{i + 1,k - \tfrac{1}{2}}} - z_{{i,k - \tfrac{1}{2}}} }}{{\Delta x_{{i + \tfrac{1}{2}}} }}g_{{i + \tfrac{1}{2},k - \tfrac{1}{2}}}^{2} - \frac{{z_{{i + 1,k + \tfrac{1}{2}}} - z_{{i,k + \tfrac{1}{2}}} }}{{\Delta x_{{i + \tfrac{1}{2}}} }}g_{{i + \tfrac{1}{2},k + \tfrac{1}{2}}}^{1} \\ A_{{i + \tfrac{1}{2},k}}^{4} & = \frac{{z_{{i + 1,k - \tfrac{1}{2}}} - z_{{i,k - \tfrac{1}{2}}} }}{{\Delta x_{{i + \tfrac{1}{2}}} }}g_{{i + \tfrac{1}{2},k - \tfrac{1}{2}}}^{3} \,\,,\,\,\,\,\,\,\,\,\,\,\,\,\,\,\,\,\,\,A_{{i + \tfrac{1}{2},k}}^{6} = - \frac{{z_{{i + 1,k + \tfrac{1}{2}}} - z_{{i,k + \tfrac{1}{2}}} }}{{\Delta x_{{i + \tfrac{1}{2}}} }}g_{{i + \tfrac{1}{2},k + \tfrac{1}{2}}}^{4} \\ A_{{i + \tfrac{1}{2},k}}^{5} & = \frac{{d_{i + 1,k} }}{{\Delta x_{{i + \tfrac{1}{2}}} }} + \frac{{z_{{i + 1,k - \tfrac{1}{2}}} - z_{{i,k - \tfrac{1}{2}}} }}{{\Delta x_{{i + \tfrac{1}{2}}} }}g_{{i + \tfrac{1}{2},k - \tfrac{1}{2}}}^{4} - \frac{{z_{{i + 1,k + \tfrac{1}{2}}} - z_{{i,k + \tfrac{1}{2}}} }}{{\Delta x_{{i + \tfrac{1}{2}}} }}g_{{i + \tfrac{1}{2},k + \tfrac{1}{2}}}^{3} \\ \end{aligned}$$

26 $$\begin{aligned} B_{{i,k + \tfrac{1}{2}}}^{1} & = - \frac{{d_{{i - \tfrac{1}{2},k + \tfrac{1}{2}}} }}{{\Delta x_{i} }}g_{{i - \tfrac{1}{2},k + \tfrac{1}{2}}}^{1} ,\,\,\,\,B_{{i,k + \tfrac{1}{2}}}^{2} = - \frac{{d_{{i - \tfrac{1}{2},k + \tfrac{1}{2}}} }}{{\Delta x_{i} }}g_{{i - \tfrac{1}{2},k + \tfrac{1}{2}}}^{2} \\ B_{{i,k + \tfrac{1}{2}}}^{3} & = \frac{{z_{{i + \tfrac{1}{2},k}} - z_{{i - \tfrac{1}{2},k}} }}{{\Delta x_{i} }} - \frac{{d_{{i - \tfrac{1}{2},k + \tfrac{1}{2}}} }}{{\Delta x_{i} }}g_{{i - \tfrac{1}{2},k + \tfrac{1}{2}}}^{3} + \frac{{d_{{i + \tfrac{1}{2},k + \tfrac{1}{2}}} }}{{\Delta x_{i} }}g_{{i + \tfrac{1}{2},k + \tfrac{1}{2}}}^{1} \\ B_{{i,k + \tfrac{1}{2}}}^{4} & = - \frac{{z_{{i + \tfrac{1}{2},k + 1}} - z_{{i - \tfrac{1}{2},k + 1}} }}{{\Delta x_{i} }} - \frac{{d_{{i - \tfrac{1}{2},k + \tfrac{1}{2}}} }}{{\Delta x_{i} }}g_{{i - \tfrac{1}{2},k + \tfrac{1}{2}}}^{4} + \frac{{d_{{i + \tfrac{1}{2},k + \tfrac{1}{2}}} }}{{\Delta x_{i} }}g_{{i + \tfrac{1}{2},k + \tfrac{1}{2}}}^{2} \\ B_{{i,k + \tfrac{1}{2}}}^{5} & = \frac{{d_{{i + \tfrac{1}{2},k + \tfrac{1}{2}}} }}{{\Delta x_{i} }}g_{{i + \tfrac{1}{2},k + \tfrac{1}{2}}}^{3} ,\,\,\,\,B_{{i,k + \tfrac{1}{2}}}^{6} = \frac{{d_{{i + \tfrac{1}{2},k + \tfrac{1}{2}}} }}{{\Delta x_{i} }}g_{{i + \tfrac{1}{2},k + \tfrac{1}{2}}}^{4} \\ \end{aligned}$$

27 $$S_{{i,k + \tfrac{1}{2}}}^{x} = \frac{{z_{{i + \tfrac{1}{2},k + \tfrac{1}{2}}} - z_{{i - \tfrac{1}{2},k + \tfrac{1}{2}}} }}{{\Delta x_{i} }}\,\,\,,\,\,\,\,\,\,\,\,\,\,\,\,\,\,\,\,\,\,\,\,\,\,S_{{i,k - \tfrac{1}{2}}}^{x} = \frac{{z_{{i + \tfrac{1}{2},k - \tfrac{1}{2}}} - z_{{i - \tfrac{1}{2},k - \tfrac{1}{2}}} }}{{\Delta x_{i} }}$$

By expressing Eq. (24) for cells, the system of linear equations involving dynamic pressure correction variables was obtained as follows:

28 $$A \cdot \Delta q = b$$

Here, *A* represents a sparse matrix of coefficients with dimensions $\left( {N_{x} \times N_{z} } \right) \times \left( {N_{x} \times N_{z} } \right)$, where *N*_*x*_ denotes the count of grid cells in the horizontal direction, *N*_*z*_ represents the count of vertical layers. *∆q* and *b* are the vectors of unknowns and knowns, respectively. Almost all computational costs of the model are related to solving Eq. (28). Therefore, the discretization method of equations and the selection of an appropriate solution technique for it have a significant impact on reducing these costs. Due to the novel discretization applied in Eq. (24), each computational cell became associated with only its nine neighboring cells. The primary difference in the current model, resulting in Eq. (24), compared to other models, is the method used to calculate horizontal velocities at locations of vertical velocities. The conventional approach for computing these velocities involves averaging the four horizontal velocities around each point. This procedure leads to the Poisson equation, wherein the calculation of each cell is dependent on 15 neighboring cells^47,48^. In contrast, Eq. (24) applies Eq. (20) in vertical velocity locations and is, therefore, derived by exclusively averaging explicit terms, reducing the dependency from 15 to 9 and resulting in a sparser matrix compared to other models. It is evident that Eq. (24) only considers the existing cells (6 adjacent cells) at the seaward boundary. This reduction in dependencies resulted in a sparser coefficient matrix and consequently played a significant role in reducing the computational cost. In this study, the linear system defined by Eq. (28) was fully implicitly resolved by employing the bi-conjugate gradient method proposed by Van der Vorst^49^. Since preconditioning techniques can expedite the convergence of the unknowns, the bi-conjugate gradient method, along with an appropriate preconditioner, was employed in this study. Various preconditioning methods exist, and one of the well-known ones is the Modified Incomplete LU Factorization (MILU)^50^. Although this preconditioner is very efficient for most systems, it incurs a high computational cost, and implementing it for parallel processing is challenging and less efficient. Additionally, when the gradient of the dynamic pressure correction is weak, its convergence is so rapid that the use of a powerful preconditioner like MILU is not necessary. Therefore, in such cases, a weaker preconditioner can be used. The MILUD preconditioner (Modified Incomplete LU factorization restricted to Diagonal) is a suitable option with lower computational costs compared to MILU. Furthermore, parallel processing can be implemented when employing the MILUD preconditioner.

After calculating the pressure correction variables, $u$ is computed using Eq. (22), $w$ is obtained from Eq. (23), and $\eta$ is determined using Eq. (19) at the subsequent time step. Additionally, the dynamic pressure at the subsequent time step is obtained from $q^{n + 1} = q^{n} + \Delta q$.

To ensure a stable solution, the Courant stability condition was employed in the form of the following equation to select the time step:

29 $$Cr=\frac{\Delta t\left(\left|{u}_{i+1/2}^{n+1}\right|+\sqrt{g.{\widehat{d}}_{i+1}^{n+1}}\right)}{\Delta x}\le 1$$

Here, *Cr* represents the Courant number. Indeed, meeting this condition ensures the model's stability. However, due to the variation in velocity and depth values across different locations during model execution, the Courant number is practically constrained to the range of 0.3 ≤ *Cr* ≤ 0.8, and this restriction was applied to all domain elements throughout the execution.

### Boundary conditions

The boundary condition at the shoreline is of the wall type, specified by imposing zero velocity at the boundary. However, it's worth noting that due to the sloping bed and the implementation of the wet/dry method, waves never reach the terminal computational cells at the shoreline. In dry areas, both velocity and pressure values are zero. Generally, the seaward boundary generation process follows these steps: utilizing wave theory equations (linear, nonlinear, solitary, etc.), the free surface elevation is initially calculated at each time step, followed by the determination of the depth and thickness of boundary cells. Subsequently, using the same wave theory equations, the horizontal velocity values at the center of each cell are also calculated. Finally, the flux values at the boundary are determined by multiplying the thickness of each cell by its velocity and then applied to the equations.

### Wave breaking simulation

In general, the mentioned equations are adept at simulating the propagation of nonlinear waves beyond the coastal region. For the propagation of weak nonlinear waves in intermediate to shallow waters, where pressure gradients and particle velocities are low, using a grid with 1 to 3 vertical layers can achieve sufficient accuracy. However, with decreasing depth and wave shoaling, as investigated in the current research, the nonlinear property increases, simultaneously intensifying vertical gradients in particle velocity. This may result in some inaccuracies in simulations using the outlined equations.

Several solutions have been proposed to enhance the accuracy of non-hydrostatic models in circumstances with high vertical gradients for detecting wave breaking locations and estimating associated energy dissipation. In Smit et al.^21^'s numerical model, a method was suggested to simulate the propagation of strong nonlinear waves in coastal areas using a grid with fewer than 5 layers. According to their approach, the onset of breaking is initially identified, then assuming a hydrostatic pressure distribution and removing vertical accelerations prevent water level rise in that area. The method proposed by Smit et al.^21^ has been incorporated into the current model, resulting in modifications to the solution of governing equations. According to this method, waves commence breaking when the rate of water level rise ($\tfrac{\partial \eta }{{\partial t}}$) surpasses the product of a steepness coefficient (*α*) and the wave speed (*c*). By enforcing this condition in each cell, a hydrostatic pressure distribution is applied (*q* = 0), and the vertical momentum equation is not solved (*w* = 0). The value of *α* depends on the dynamic wave conditions and the vertical resolution used in the model, such that increasing the number of layers increases the value of *α*. After identifying the location of the wave breaking, it is necessary to dissipate the energy generated by the wave breaking or to prevent the wave from further cresting. Among the available methods for this purpose, the most suitable is the Hydrostatic Front Approximation (HFA) method^51^.

In this study, to examine the impact of the HFA method, numerical simulations were conducted both with and without the implementation of the method, and subsequently, the results were compared.

### Validation of the numerical model

**Figure 3:**
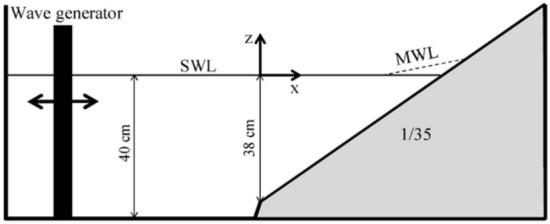
The bed shape and coordinate system for the breaking of reugular wave on a sloping beach^52^.

The current non-hydrostatic model aims to estimate the distribution of parameters such as velocity and kinetic energy in depth. To evaluate the intended objective, the Ting and Kirby^52^ test was utilized. The experimental tests by Ting and Kirby^52^ were related to spilling and plunging breakers. Figure 3 provides a schematic view of the flume in these experiments along with the coordinate system.

In all experiments, the still water depth in the constant-depth region of the wave tank was 0.4 m, and Cnoidal waves were generated. For the spilling and plunging breaker, wave heights of 1.25 m and 1.28 m, and wave periods of 2 s and 5 s, respectively, were considered. These were the input wave characteristics at the location x = − 10 m concerning the starting point of the slope. In the simulation of these tests, discharge values, modified with return fluxes, were specified at each layer at the generating boundary based on second order Cnoidal waves^53^.

For the computational mesh, a horizontal grid size of *∆x* = 0.025 m was used. To accurately capture parameters in the depth, 16 layers with equal thickness were considered. The simulation duration was 120 s, and the time step (*∆t*) was set to 0.0025 s.

**Figure 4:**
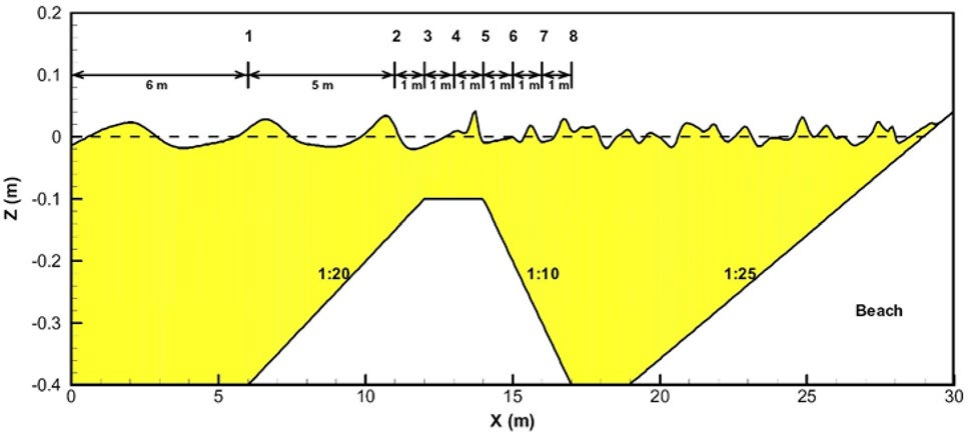
Defenition sketch of wave flume and locations of wave gauge^54^.

Following the assessment of the model's precision in estimating parameters at depth, it was employed to enhance the findings of the Beji and Battjes^54^ experiments. Initially, the numerical model underwent validation using the data reported in the Beji and Battjes^54^ study. Subsequently, it was utilized to determine velocity values at various depths. Notably, due to the absence of velocity measurements in the Beji and Battjes^54^ study, evaluating the accuracy of the obtained profiles was unfeasible, and these profiles were solely provided to augment the experimental study conducted by Beji and Battjes^54^. The experimental configuration is depicted in Fig. 4. The submerged bar profile includes an upslope with a slope ratio of 1:20, a horizontal crest extending 2 m with a still water depth of 0.10 m, succeeded by a downslope with a slope ratio of 1:10. In areas of deep water, the still water depth is maintained at 0.40 m. Eight wave gauges, denoted as WG1, WG2, …, WG8, were employed to measure the waves. The study encompassed long periodic waves with a frequency of approximately 0.4 Hz, as well as short periodic regular waves with a frequency of around 1.0 Hz.

## Results and discussion

In the Ting and Kirby^52^ test, in addition to parameters such as water surface elevation (wave height, mean water surface level, and wave profile), other parameters like turbulent kinetic energy (*k*) and horizontal velocity at various locations were also presented as time-averaged distributions at depth in both spilling and plunging breaker cases. Considering that the tests exhibit significant nonlinear properties and very severe wave breaking, few have sought to validate their numerical models with these tests. Most models that have utilized these tests have focused on estimating parameters related to the water surface elevation^22,23^. Specially, models based on solving the water surface elevation (VOF models) have primarily used these tests for validation^8–10^. However, it should be noted that this does not imply that non-hydrostatic models are incapable of estimating depth-dependent parameters such as velocity distributions. To illustrate this assertion, the existing numerical model was employed for the application of the Ting and Kirby^52^ test in both breaking cases, to estimate the distribution of parameters within the depth.

**Figure 5:**
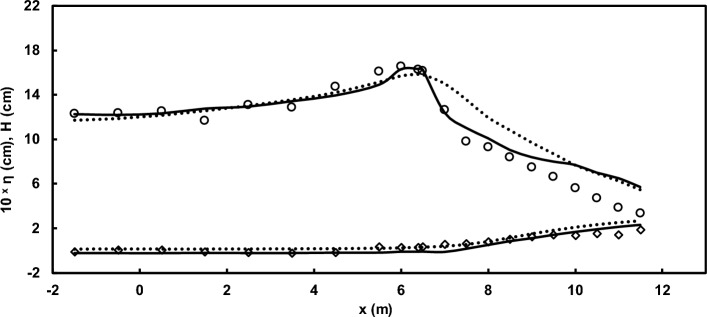
Comparison of wave height (upper trend) and set-up (lower trend) in the spilling breaker scenario between the Ting and Kirby^52^ experimental data (symbols) and results of the current model with and without using HFA method (solid line and dotted line).

**Figure 6:**
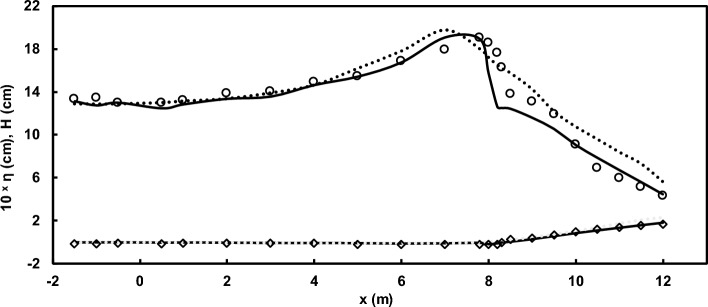
Comparison of wave height (upper trend) and set-up (lower trend) in the plunging breaker scenario between the Ting and Kirby^52^ experimental data (symbols) and results of the current model with and without using HFA method (solid line and dotted line).

In this article, initially, to showcase the proficiency of the developed model in estimating water surface elevation and wave height, a comparison was drawn between measurements from the laboratory model by Ting and Kirby^52^ and results from the current numerical model. The findings underscored the effectiveness of the current numerical model in estimating these parameters (Figs. 5 and 6). It is essential to note that the governing equations are solved and discretized using a conservative approach. When there is a high number of layers and factors such as diffusion resulting from eddy viscosity and bed roughness are taken into account, wave breaking, akin to a discontinuity in the water surface elevation, can be resolved, leading to energy dissipation. However, the precise location of breaking and energy dissipation after breaking does not align well with experimental results. Hence, it becomes essential to employ a mechanism to identify the breaking location and energy dissipation accurately.

Subsequently, the model's capability in estimating parameters at depth was assessed. The model output was extracted as a time series of turbulent kinetic energy (*k*) and horizontal velocity (*u*) before and after the breaking point at each layer in the depth. For aligning the numerical model results with laboratory data, the values of *u* and *k* obtained on the Lagrangian grid were first transformed to a fixed-grid or Eulerian grid using linear interpolation. Then, by temporally averaging these parameters, the average values of turbulent kinetic energy ($\overline{k}$) and horizontal velocity (*ū*) were calculated. Finally, to conform to the laboratory data, the values of *z* were normalized using the $(z - \overline{\eta })/\overline{h}$, *ū* were normalized using the $\overline{u}/\sqrt {g\overline{h}}$ , and $\overline{k}$ were normalized using the $\sqrt {\overline{k}/g\overline{h}}$ .

**Figure 7:**
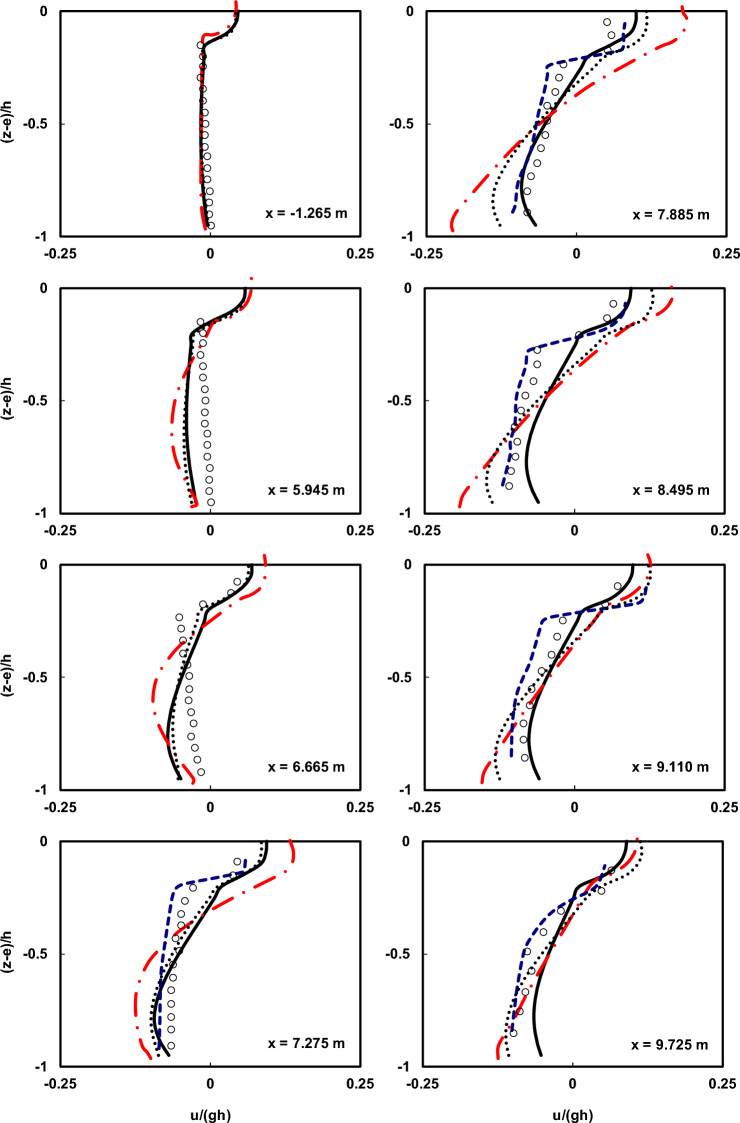
Comparison of the normalized average horizontal velocity profiles of the undertow flow at different locations both before and after breaking point in the spilling breaker scenario between the Ting and Kirby^52^ experimental data (circles), results of the current model with and without using HFA method (solid line and dotted line), results of the Bakhtyar et al.^8^ model (dashed line), and results of the Derakhti et al.^25^ model (dash-dot line).

**Figure 8:**
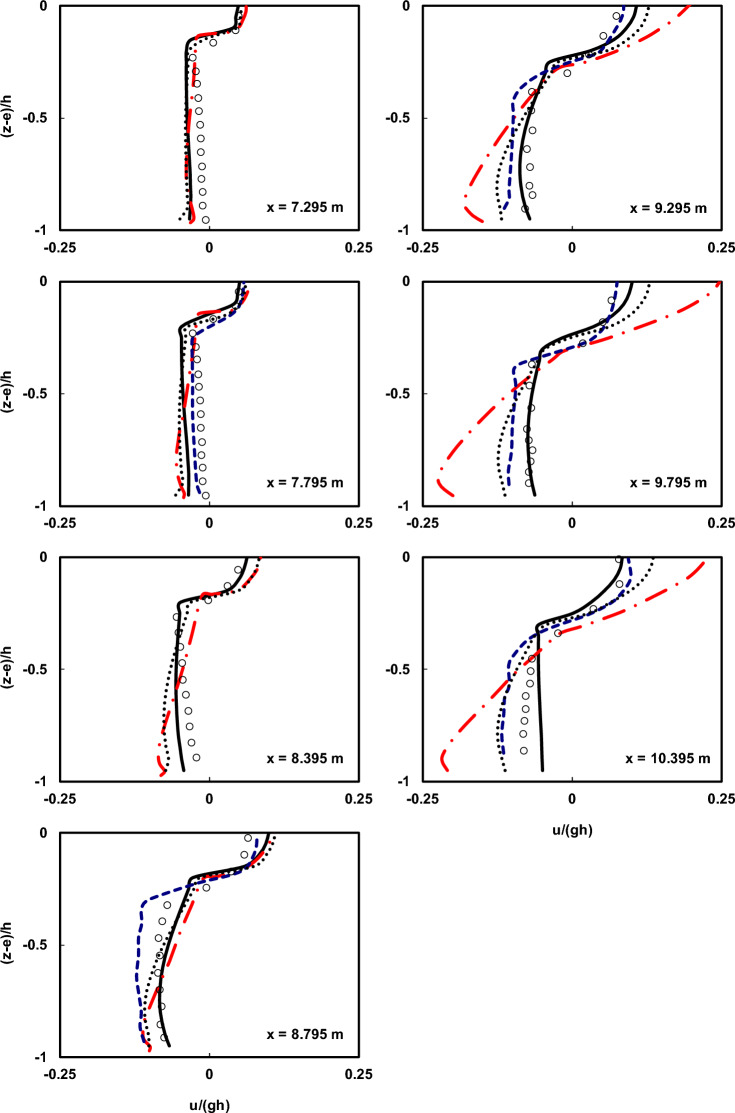
Comparison of the normalized average horizontal velocity profiles of the undertow flow at different locations both before and after breaking point in the plunging breaker scenario between the Ting and Kirby^52^ experimental data (circles), results of the current model with and without using HFA method (solid line and dotted line), results of the Bakhtyar et al.^8^ model (dashed line), and results of the Derakhti et al.^25^ model (dash-dot line).

Figures 7 and 8 depict a comparison of the depth-wise distribution of average velocities between the Ting and Kirby^52^ laboratory data and the outcomes of the current numerical model at various locations for spilling and plunging breaker cases, respectively. To assess the influence of the HFA approach, numerical simulations were carried out with and without utilizing the method. In all the figures presented regarding the utilization of the HFA method, the results for spilling breakers were acquired with *α* set to 1.4, and those for plunging breakers were obtained with *α* set to 2.2. Moreover, the presented figures depict the outcomes of the Bakhtyar et al.^8^ model, an example of a VOF model, and the Derakhti et al.^25^ model, which is a non-hydrostatic model.

As observed in the figures, the estimated undertow flow profiles by the current model conform well to the measured profiles with very low computational cost (*∆x* = 0.025 m and 16 vertical layers). This is achieved while the computational cost of the Bakhtyar et al.^8^ model (*∆x* = 0.015 m and 120 vertical layers) is significantly higher than the current model, and its better performance is attributed to the use of a finer grid and the utilization of the free-surface VOF method. However, the results obtained from the current model are satisfactory both before and well after the breaking point, with only slight discrepancies near the breaking point. Certainly, the main reason for this difference lies in the way non-hydrostatic models handle the water surface elevation, calculating it as the mass continuity of the water column, which cannot capture the complexities of water surface elevation as VOF-based models do. Furthermore, as mentioned earlier, another factor contributing to this difference is the nature of the simulated waves, characterized by high nonlinearity and pronounced vertical gradients in particle velocity. The findings suggest that using the HFA method helps alleviate this disparity. Moreover, contrasting the results with those derived from the non-hydrostatic model developed by Derakhti et al.^25^, underscores the enhanced proficiency of the present numerical model.

**Figure 9:**
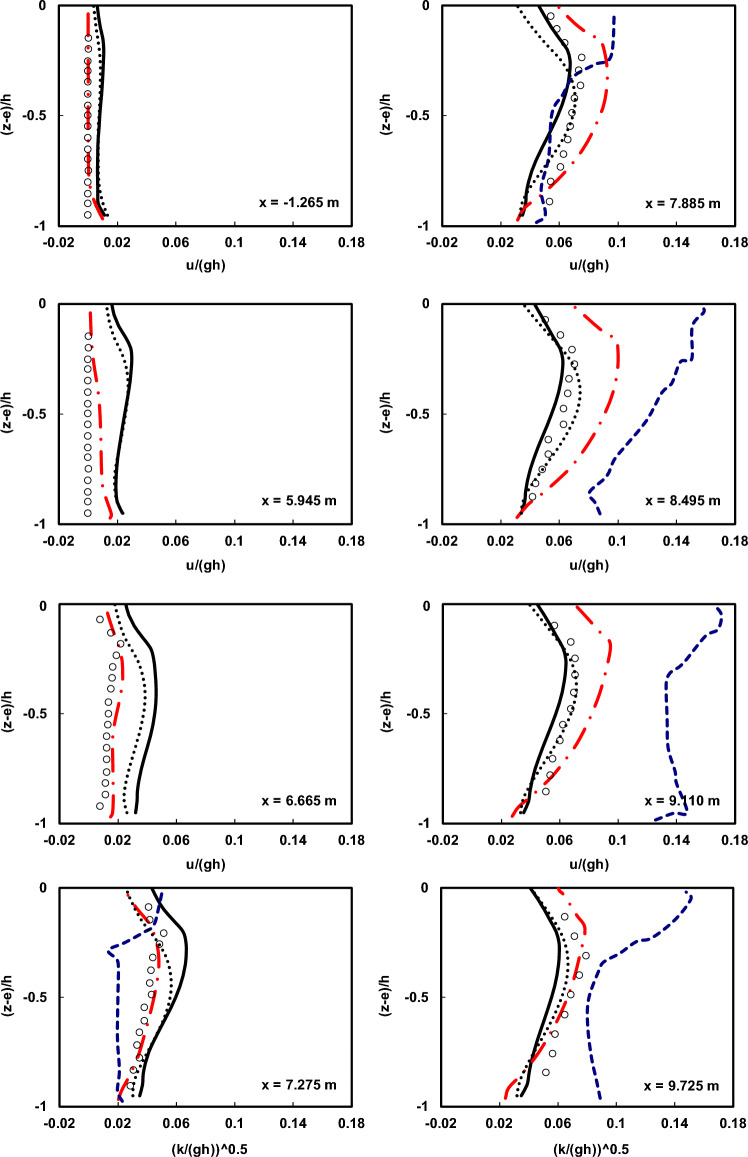
Comparison of the normalized average turbulent kinetic energy profiles of the undertow flow at different locations both before and after breaking point in the spilling breaker scenario between the Ting and Kirby^52^ experimental data (circles), results of the current model with and without using HFA method (solid line and dotted line), results of the Bakhtyar et al.^8^ model (dashed line), and results of the Derakhti et al.^25^ model (dash-dot line).

**Figure 10:**
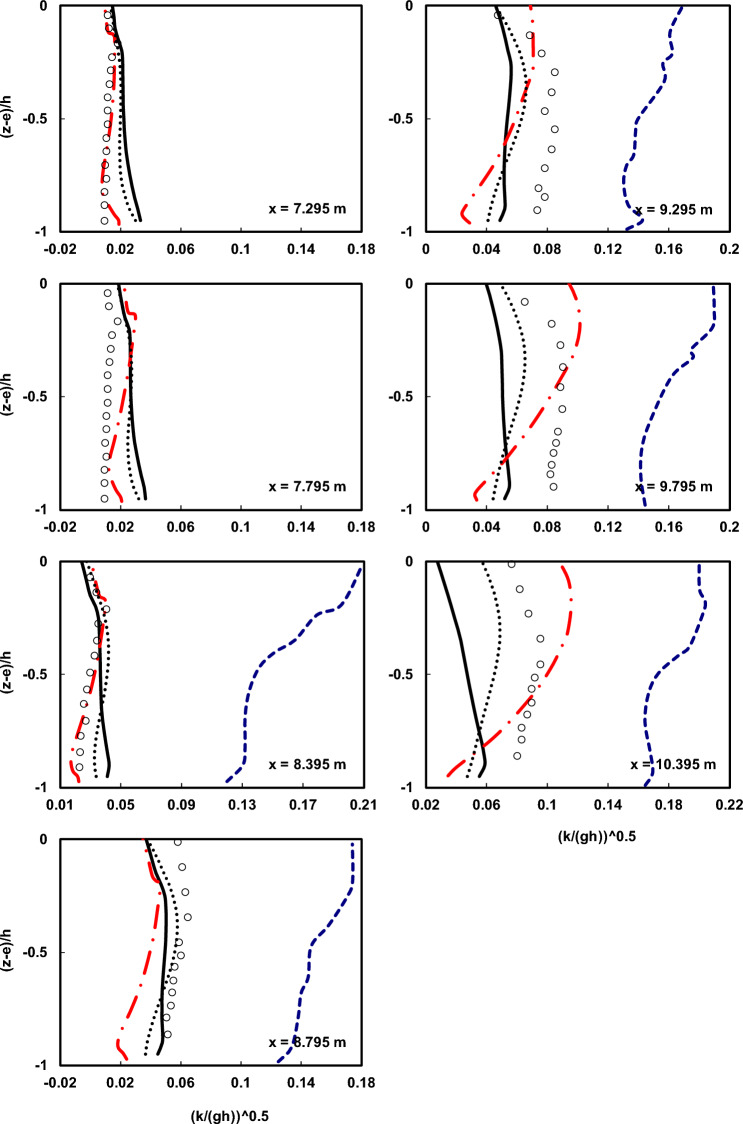
Comparison of the normalized average turbulent kinetic energy profiles of the undertow flow at different locations both before and after breaking point in the plunging breaker scenario between the Ting and Kirby^52^ experimental data (circles), results of the current model with and without using HFA method (solid line and dotted line), results of the Bakhtyar et al.^8^ model (dashed line), and results of the Derakhti et al.^25^ model (dash-dot line).

Figures 9 and 10 compare the calculated mean kinetic energy ($\overline{k}$) by the numerical model, respectively, for plunging and spilling breakers with the laboratory data from Ting and Kirby^52^ and the results of the Bakhtyar et al.^8^ model at various locations.

As evident from the results, the estimated $\overline{k}$ profile by the developed numerical model shows much better agreement both in terms of shape and magnitude compared to the Bakhtyar et al.^8^ model with the experimental data. According to theory, the higher the turbulent kinetic energy, the higher the turbulent viscosity coefficient (*ν*_*t*_) becomes, resulting in a more uniform velocity profile in the depth. Therefore, the factor influencing the uniformization of the velocity profile in depth is the distribution of the turbulent viscosity coefficient (*ν*_*t*_). In contrast, wave-induced forces such as gradients in radiation stress and mean water surface elevation lead to the generation of undertow and curvature in the velocity profile. The balance between these two factors leads to the creation of an accurate velocity profile.

**Figure 11:**
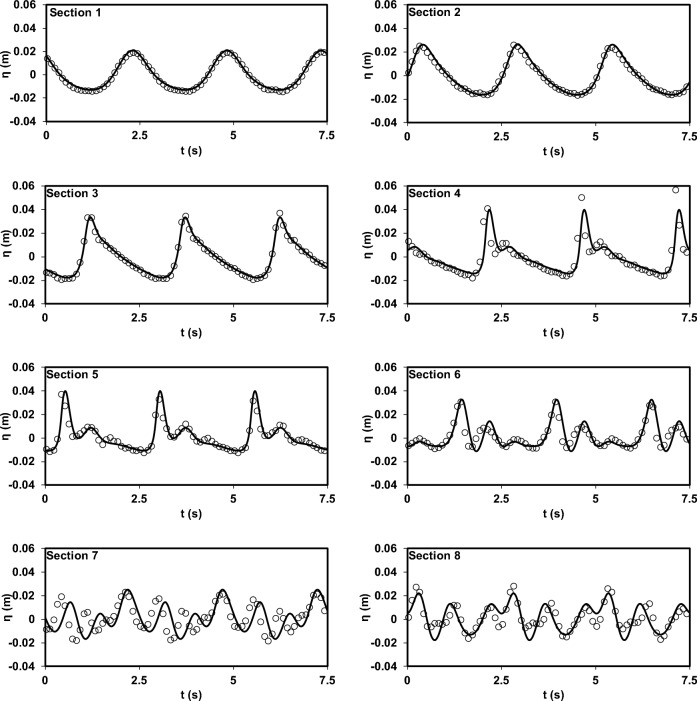
Comparison of water surface elevation in the spilling breaker scenario between the Beji and Battjes^54^ experimental data (circles) and results of the current model (solid line).

**Figure 12:**
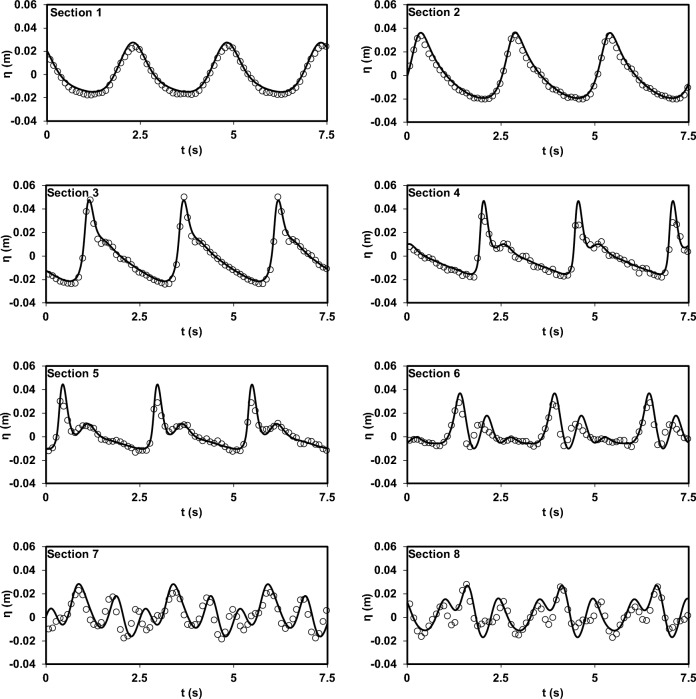
Comparison of water surface elevation in the plunging breaker scenario between the Beji and Battjes^54^ experimental data (circles) and results of the current model (solid line).

**Figure 13:**
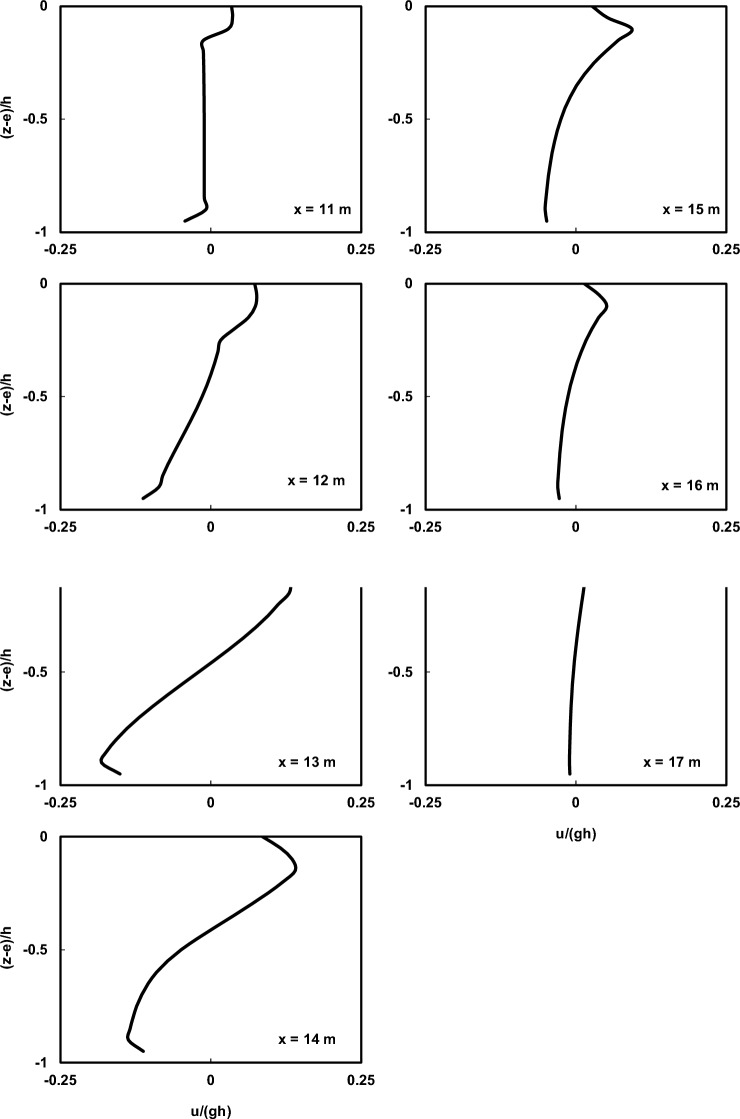
Prediction of normalized average horizontal velocity profiles at various locations in the spilling breaker scenario of the Beji and Battjes^54^ experiment by the current model.

**Figure 14:**
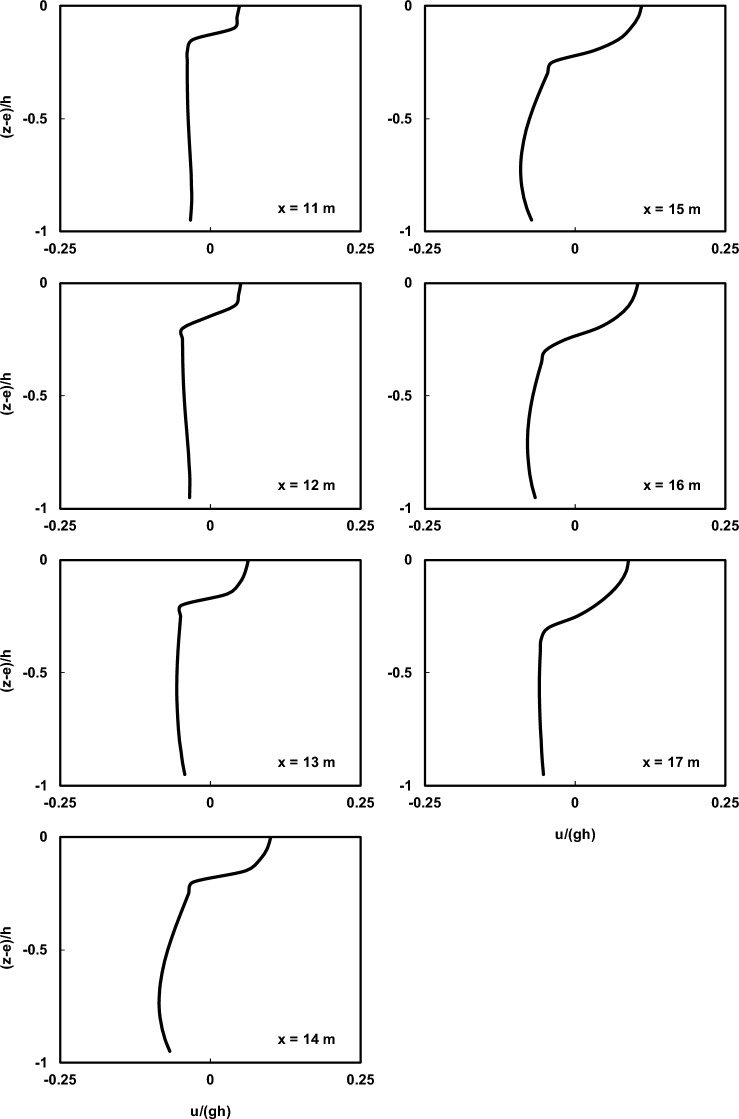
Prediction of normalized average horizontal velocity profiles at various locations in the plunging breaker scenario of the Beji and Battjes^54^ experiment by the current model.

As mentioned earlier, for a comprehensive evaluation of the model's capabilities, the results of the experimental study by Beji and Battjes^54^ were utilized. This study is notable for its significant dispersion conditions. Initially, the model's accuracy in predicting the water surface elevation, as reported in the research, was evaluated for both spilling and plunging breaker scenarios (Figs. 11 and 12). As depicted in the figures, the model demonstrates a remarkable ability to reproduce the experimental data. Subsequently, after this validation, the model was employed to complement the experimental findings of Beji and Battjes^54^ by predicting horizontal velocity profiles at different depths (Figs. 13 and 14).

## Conclusion

In the current research, a non-hydrostatic model was developed to estimate the distribution of hydraulic parameters in depth before and after the wave breaking point. The numerical approach employed in the present study was the projection method with a pressure-correction technique, incorporating the elimination of velocities and a time splitting technique for various solution phases. In the current model, modifications were made to the governing equations and the solution approach to locally solve monotonicity and momentum conservation. Accordingly, the momentum equations were simultaneously solved with the mass equation. Considering the role of the horizontal advection term of horizontal velocities in simulating wave propagation in shallow water, especially during wave breaking, a shock-capturing method was utilized to solve this term. The Poisson pressure correction equation was also discretized using a novel method, associating the calculation of each cell with only its nine neighboring cells.

The simulated experimental tests by this model were undertow tests, in which the model's capability to estimate variables like turbulent kinetic energy and velocity in depth was evaluated. The results showed that this non-hydrostatic model effectively reproduces the distribution of parameters in depth. Therefore, the developed model demonstrates the capability to satisfactorily perform simulations for phenomena where the distribution of parameters in depth is required, all with low computational costs.

## Data Availability

The datasets used and/or analyzed during the current study available from the corresponding author on reasonable request.

## References

[1] Munk WH (1949). The solitary wave theory and its application to surf problems. Ann. N. Y. Acad. Sci..

[2] Stokes GG (1880). Supplement to a paper on the theory of oscillatory waves. Math. Phys. Pap..

[3] Michell, J. H. XLIV. The highest waves in water. *London, Edinburgh, Dublin Philos. Mag. J. Sci.* **36**, 430–437 (1893).

[4] Miche, M. Mouvements ondulatoires de la mer en profondeur constante ou décroissante. *Ann. Ponts Chaussées, *26–78, (2) 270–292, (3) 369–406 (1944).

[5] Kamphuis JW (1991). Incipient wave breaking. Coast. Eng..

[6] Battjes JA, Janssen J (1978). Energy loss and set-up due to breaking of random waves. Coast. Eng..

[7] Dally WR, Dean RG, Dalrymple RA (1985). Wave height variation across beaches of arbitrary profile. J. Geophys. Res. Ocean..

[8] Bakhtyar R, Barry DA, Yeganeh-Bakhtiary A, Ghaheri A (2009). Numerical simulation of surf-swash zone motions and turbulent flow. Adv. Water Resour..

[9] Li Y, Larsen BE, Fuhrman DR (2022). Reynolds stress turbulence modelling of surf zone breaking waves. J. Fluid Mech..

[10] Zhao Q, Armfield S, Tanimoto K (2004). Numerical simulation of breaking waves by a multi-scale turbulence model. Coast. Eng..

[11] Chella MA, Bihs H, Myrhaug D, Muskulus M (2016). Hydrodynamic characteristics and geometric properties of plunging and spilling breakers over impermeable slopes. Ocean Model..

[12] Derakhti M, Kirby JT, Shi F, Ma G (2016). NHWAVE: Consistent boundary conditions and turbulence modeling. Ocean Model..

[13] Lin P, Liu PL-F (1998). A numerical study of breaking waves in the surf zone. J. Fluid Mech..

[14] Zijlema M, Stelling GS (2008). Efficient computation of surf zone waves using the nonlinear shallow water equations with non-hydrostatic pressure. Coast. Eng..

[15] Zijlema M, Stelling GS (2005). Further experiences with computing non-hydrostatic free-surface flows involving water waves. Int. J. Numer. Methods Fluids.

[16] Stelling GS, Duinmeijer SPA (2003). A staggered conservative scheme for every Froude number in rapidly varied shallow water flows. Int. J. Numer. Methods Fluids.

[17] Yamazaki Y, Kowalik Z, Cheung KF (2009). Depth-integrated, non-hydrostatic model for wave breaking and run-up. Int. J. Numer. Methods Fluids.

[18] Ai C, Jin S (2012). A multi-layer non-hydrostatic model for wave breaking and run-up. Coast. Eng..

[19] Moghadam KF, Banihashemi MA, Badiei P, Shirkavand A (2019). A numerical approach to solve fluid-solid two-phase flows using time splitting projection method with a pressure correction technique. Prog. Comput. Fluid Dyn. Int. J..

[20] Moghadam KF, Banihashemi MA, Badiei P, Shirkavand A (2020). A time-splitting pressure-correction projection method for complete two-fluid modeling of a local scour hole. Int. J. Sediment Res..

[21] Smit P, Zijlema M, Stelling G (2013). Depth-induced wave breaking in a non-hydrostatic, near-shore wave model. Coast. Eng..

[22] Shirkavand A, Badiei P (2014). The application of a Godunov-type shock capturing scheme for the simulation of waves from deep water up to the swash zone. Coast. Eng..

[23] Derakhti M, Kirby JT, Shi F, Ma G (2016). Wave breaking in the surf zone and deep-water in a non-hydrostatic RANS model. Part 1: Organized wave motions. Ocean Model..

[24] Iravani N, Badiei P, Brocchini M (2020). Novel free surface boundary conditions for spilling breaking waves. Coast. Eng..

[25] Derakhti M, Kirby JT, Shi F, Ma G (2016). Wave breaking in the surf zone and deep-water in a non-hydrostatic RANS model. Part 2: Turbulence and mean circulation. Ocean Model..

[26] Kazolea M, Ricchiuto M (2018). On wave breaking for Boussinesq-type models. Ocean Model..

[27] Papoutsellis CE, Yates ML, Simon B, Benoit M (2019). Modelling of depth-induced wave breaking in a fully nonlinear free-surface potential flow model. Coast. Eng..

[28] Simon B, Papoutsellis CE, Benoit M, Yates ML (2019). Comparing methods of modeling depth-induced breaking of irregular waves with a fully nonlinear potential flow approach. J. Ocean Eng. Mar. Energy.

[29] Tatlock B, Briganti R, Musumeci RE, Brocchini M (2018). An assessment of the roller approach for wave breaking in a hybrid finite-volume finite-difference Boussinesq-type model for the surf-zone. Appl. Ocean Res..

[30] Kennedy AB, Chen Q, Kirby JT, Dalrymple R (2000). A. Boussinesq modeling of wave transformation, breaking, and runup. I: 1D. J. Waterw. Port Coast. Ocean Eng..

[31] Cienfuegos R, Barthélemy E, Bonneton P (2010). Wave-breaking model for Boussinesq-type equations including roller effects in the mass conservation equation. J. Waterw. Port Coast. Ocean Eng..

[32] Dutykh D, Katsaounis T, Mitsotakis D (2011). Finite volume schemes for dispersive wave propagation and runup. J. Comput. Phys..

[33] Khakimzyanov G, Dutykh D, Gusev O, Shokina N (2018). Dispersive shallow water wave modelling. Part II: Numerical simulation on a globally flat space. Commun. Comput. Phys..

[34] Abdalazeez A, Didenkulova II, Dutykh D, Denissenko P (2020). Comparison of dispersive and nondispersive models for wave run-up on a beach. Izv. Atmos. Ocean. Phys..

[35] Pilloton C, Lugni C, Graziani G, Fedele F (2023). Wave dispersion in moderate channel turbulence. Sci. Rep..

[36] Smagorinsky J (1963). General circulation experiments with the primitive equations: I. The basic experiment. Mon. Weather Rev..

[37] Shirkavand A, Badiei P (2015). Evaluation and modification of time splitting method applied to the fully dynamic numerical solution of water wave propagation. Prog. Comput. Fluid Dyn. Int. J..

[38] Namin MM (2003). A Fully Three-Dimensional Non-hydrostatic Free Surface Flow Model for Hydro-Environmental Predictions: Numerical Investigations and Development of a Fully Three-Dimensional Hydrodynamic (Non-hydrostatic) Turbulence and Solute Transport Model Based on an.

[39] Easter RC (1993). Two modified versions of Bott’s positive-definite numerical advection scheme. Mon. Weather Rev..

[40] Ruddick, K. G. Modelling of coastal processes influenced by the freshwater discharge of the Rhine (1998).

[41] Wu Y, Falconer RA (2000). A mass conservative 3-D numerical model for predicting solute fluxes in estuarine waters. Adv. Water Resour..

[42] Pietrzak J, Jakobson JB, Burchard H, Vested HJ, Petersen O (2002). A three-dimensional hydrostatic model for coastal and ocean modelling using a generalised topography following co-ordinate system. Ocean Model..

[43] Lee JW, Teubner MD, Nixon JB, Gill PM (2006). A 3-D non-hydrostatic pressure model for small amplitude free surface flows. Int. J. Numer. Methods Fluids.

[44] Javan M, Namin MM, Neyshabouri SAAS (2007). A time-splitting method on a nonstaggered grid in curvilinear coordinates for implicit simulation of non-hydrostatic free-surface flows. Can. J. Civ. Eng..

[45] Toro EF (2001). Shock-Capturing Methods for Free-Surface Shallow Flows.

[46] Zijlema M, Stelling G, Smit P (2011). SWASH: An operational public domain code for simulating wave fields and rapidly varied flows in coastal waters. Coast. Eng..

[47] Ahmadi A, Badiei P, Namin MM (2007). An implicit two-dimensional non-hydrostatic model for free-surface flows. Int. J. Numer. Methods Fluids.

[48] Badiei P, Namin MM, Ahmadi A (2008). A three-dimensional non-hydrostatic vertical boundary fitted model for free-surface flows. Int. J. Numer. Methods fluids.

[49] Van der Vorst HA (1992). Bi-CGSTAB: A fast and smoothly converging variant of Bi-CG for the solution of nonsymmetric linear systems. SIAM J. Sci. Stat. Comput..

[50] Chan, T. F. & Van Der Vorst, H. A. Approximate and incomplete factorizations. in *Parallel Numerical Algorithms* 167–202 (Springer, 1997).

[51] Tonelli M, Petti M (2010). Finite volume scheme for the solution of 2D extended Boussinesq equations in the surf zone. Ocean Eng..

[52] Ting FCK, Kirby JT (1994). Observation of undertow and turbulence in a laboratory surf zone. Coast. Eng..

[53] Horikawa K (1988). Nearshore Dynamics and Coastal Processes: Theory, Measurement, and Predictive Models.

[54] Beji S, Battjes JA (1993). Experimental investigation of wave propagation over a bar. Coast. Eng..

